# Refining a questionnaire to assess breast cancer knowledge and barriers to screening in Kenya: Psychometric assessment of the BCAM

**DOI:** 10.1186/s12913-017-2058-x

**Published:** 2017-02-03

**Authors:** J. Wachira, A. Busakhala, F. Chite, V. Naanyu, J. Kisuya, G. Otieno, A. Keter, A. Mwangi, T. Inui

**Affiliations:** 1 Academic Model Providing Access to Healthcare (AMPATH) Partnership, P.O Box 4604–30100, Eldoret, Kenya; 2 School of Medicine, Department of Medicine, Moi University, P.O Box 4604–30100, Eldoret, Kenya; 3 School of Medicine, Department of Behavioral Sciences, Moi University, P.O Box 4604–30100, Eldoret, Kenya; 4 School of Medicine, Indiana University, 1110 W Michigan St, Indianapolis, IN 46202 USA; 5 Regenstrief Institute, 410 W 10th St, Indianapolis, IN 46202 USA

**Keywords:** Psychometric assessment, Breast cancer, Knowledge, Barriers to screening, Kenya

## Abstract

**Background:**

Our study objective was to determine the validity and reliability of the breast module of a cancer awareness measure (BCAM) among adult women in western Kenya.

**Methods:**

The study was conducted between October and November 2012, following three breast cancer screening events. Purposive and systematic random sampling methods were used to identity 48 women for cognitive focus group discussions, and 1061 (594 who attended vs. 467 who did not attend screening events) for surveys, respectively. Face and psychometric validity of the BCAM survey was assessed using cognitive testing, factor analysis of survey data, and correlations. Internal reliability was assessed using Cronbach’s alpha.

**Results:**

Among survey participants, the overall median age was 34 (IQR: 26–44) years. Compared to those women who did not attend the screening events, women attendees were older (median: 35 vs. 32 years, *p* = 0.001) more often married (79% vs. 72%, *p* = 0.006), more educated (52% vs. 46% with more than an elementary level of education, *p* = 0.001), more unemployed (59% vs. 11%, *p* = 0.001), more likely to report doing breast self-examination (56% vs. 40%, *p* = 0.001) and more likely to report having felt a breast lump (16% vs. 7%, *p* = 0.001). For domain 1 on knowledge of breast cancer symptoms, one factor (three items) with Eigen value of 1.76 emerged for the group that did not attend screening, and 1.50 for the group that attended screening. For both groups two factors (factor 1 “internal influences” and factor 2 “external influences”) emerged among domain 4 on barriers to screening, with varied item loadings and Eigen values. There were no statistically significant differences in the factor scores between attendees and non-attendees. There were significant associations between factor scores and other attributes of the surveyed population, including associations with occupation, transportation type, and training for and practice of breast self-examination. Cronbach’s alpha showed an acceptable internal consistency.

**Conclusion:**

Certain subpopulations are less likely than others to attend breast screening in Kenya. A survey measure of breast cancer knowledge and perceived barriers to screening shows promise for use in Kenya for characterizing clinical and community population beliefs, but needs adaptation for setting, language and culture.

## Background

Among all cancers, breast cancer has the highest cancer-related morbidity and mortality rates in sub-Saharan Africa [1–4], and these rates are on the rise [2]. It is reported that 70–90% of the women affected by breast cancer in this region present with late-stage disease with poor outcomes as a result [4]. Even though approaches to enhancing early diagnosis and treatment have been advocated [3, 5], the region is faced with a number of challenges to achieving earlier diagnosis and care, including limited funds for health care services, underfunded health care facilities, lack of mammography equipment, and low levels of community awareness of breast cancer [3–7]. Taken together, these limitations have had a major adverse impact on efforts to reduce the stage at which breast cancer is diagnosed and treated.

In spite of these many challenges, screening programs that feature self and clinician breast exams as well as mammograms (where available) have been advocated as important stepping stones to promote public awareness, timely diagnosis and treatment and cancer prevention [2, 5]. Even when preventive services are available, however, community participation in these activities has been variable and limited. Breast cancer screening uptake in developed countries has been associated with factors such as being older in age, married, having a higher socio-economic status, more physician endorsement and having a higher social status [8]. Unfortunately, there is little or no comparable information in resource-scarce environments in sub-Saharan Africa. In the context of initial efforts to develop appropriate approaches to breast cancer screening in western Kenya, we felt the need to have a better understanding of the levels of public awareness, perceptions of breast cancer, and screening practices in various communities served by a health care delivery system. If a well-formed and valid survey instrument could be developed to characterize these matters, we believed that educational programs for the public could be focused to fill gaps in knowledge and perhaps stimulate greater volunteerism for screening.

Contemplating the use of a questionnaire that could be used to characterize citizen opinions of relevance to breast cancer screening, we unfortunately found no validated scales that had been field-tested in a Kenyan population. The literature however revealed that a number of validated scales had been developed for North American or European populations [8–11], scales whose psychometric properties would need to be evaluated if we were to adapt them for use in a Kenyan population. The value of assessing the psychometric properties of a scale to determine its validity and reliability within a specific cultural setting cannot be overstated [12, 13]. Cross-cultural and language differences routinely introduce measurement biases that affect the quality of data collected [12, 13].

After review of measures, we adopted a validated breast module of cancer awareness measure (BCAM), originally developed to determine level of cancer awareness and associated factors for the UK population [11]. The BCAM was attractive to us because it included measures of breast cancer awareness and perceived barriers to breast cancer screening. Our system’s oncologists considered both of these cognitive domains to constitute major impediments to timely screening for the detection of early-stage breast cancer. In UK populations, BCAM readability had been found to be high and the measure was acceptable to women. Construct validity was supported by significant differences between the levels of cancer awareness among cancer professionals compared to non-medical academics (50% vs. 6%, *p* = 0.001) attending cancer screening programs [11].

In order to the use the BCAM in western Kenya, collaborative research group (the Walther Project group) believed that new descriptive work to assess the psychometric characteristics of the BCAM should be carried out. Our overall study objectives were to assess the face validity, language appropriateness and internal reliability of the BCAM among adult women in western Kenya. We were also interested in exploratory factor analyses to discover any internal structure within the data from BCAM when administered to our catchment area populations. In psychometrics, ‘internal structure’ refers to a pattern of responses to items in a questionnaire. Items that cohere together illuminate the instrument’s dimensionality. In this communication, we report the procedures and findings of our work as a potential guide to others undertaking analogous work.

## Methods

### Study site

Although collaboration in health care, education and research had linked Indiana University School of Medicine and its Kenyan partners since 1989, the health care delivery system Academic Model Providing Access to Healthcare (AMPATH) was not formally designated until 2001 as a joint partnership among Moi University School of Medicine, the Moi Teaching and Referral Hospital, the Kenyan Ministry of Health and a consortium of North American medical schools lead by Indiana University School of Medicine [14]. The initial goal of AMPATH was to establish an HIV care delivery system to serve the needs of both urban and rural patients. The program operates now in 25 Ministry of Health facilities with numerous satellite clinics in a large geographic area of western Kenya. Over the years since 2001, AMPATH has expanded its mission to embrace primary health care and chronic disease management, including the prevention and care of cancer. The AMPATH Oncology Institute (AOI) was developed from the platform of the HIV-care program to address the care of cancer patients, for whom there were limited treatment options available. AOI has evolved over time with the first services being pediatric oncology, which transitioned into care for AIDS-related malignancies, then to broad-based cancer treatment services, and most recently, a formally structured model for rationed care commensurate with the resource constraints and population burden of western Kenya [9]. Within the AOI, the “Walther project” was initiated in 2011 when a grant was made by the Walther Cancer Foundation (“*The IU Simon Cancer Center (IUSCC), AMPATH-Oncology Institute (AOI): An Exemplar of Care for the Developing World and a Population-based Research Environment for IUSCC*”) in support of cancer research in Kenya. The Walther project has focused on cancer prevention activities and their evaluation, especially activities that respond to challenges in the AMPATH service area in western Kenya posed by breast and cervical cancer.

The Walter project personnel, working in collaboration with the AMPATH oncology team, conducted free breast cancer screening events in October-November of 2012 at three AMPATH sites – Mosoriot (one-day event), Turbo (two-day event), and Kapsokwony (two-day event). In the absence of mammography availability, the screening services offered were clinician breast examination by health care providers (physician-oncologists). All screening events were held at the Ministry of Health centers in the respective sites. One week before the events, posters, community meetings (*mabaraza*), and word-of-mouth information dissemination through community health workers were used to publicize the screening events and to invite community member participation. While the aim of the screening events was to screen otherwise healthy women, individuals who were found to have a breast mass were given a return date when biopsies could be done to determine whether they had breast cancer. Care for those with cancer was provided at the western Kenyan national referral facility in Eldoret (Moi Teaching and Referral Hospital).

### Study design

BCAM study surveys were conducted in October and November 2012, following three breast cancer screening events. The study was completed in three phases: 1) focus group cognitive interviews preceding the use of the BCAM, 2) health facility screening event participant surveys with the revised BCAM, and 3) household surveys with the revised BCAM in the catchment service areas of the health centers. We targeted women 18 years and older from the respective communities. Purposive sampling was used to identify participants (community women) for the cognitive interviews that were conducted in 6 focus group discussions (FGDs), with an average of 8 participants per group. The health facility survey included respondents who attended the screening events, while the household survey was conducted one day after the screening events and targeted community women who had not attended the screening. Any women in households who reported they had attended the previous day’s screening event were ineligible for the community survey. For the health facility-based survey, systemic random sampling was used to solicit participation among women waiting to be screened. We randomly selected the first person and thereafter every third person as they presented themselves for screening. A total of 1238 women were screened and 594 (48% of total screening attendees) consenting women were recruited for the household survey. Similarly, systematic random sampling with replacement method was used to identify the study sample for the household survey. We approached random households along all access routes that extended from each health center into its surrounding community. All 467 women recruited for the household survey, provided written consent and participated in the study. The survey research assistants, however, did not assess household census information so we are unable to directly report true community-based participation rates for women. Approval for this study was obtained from the Moi University Institutional Research and Ethics Committee (IREC) as well as the Indiana University Institutional Review Board (IRB).

### Instrument refinement

For instrument refinement, we adopted the BCAM questionnaire content that included demographic factors plus question items in 7 domains: (1) knowledge of symptoms; (2) confidence, skills and behavior in relation to breast changes; (3) anticipated delay in contacting the doctor; (4) barriers to seeking medical help; (5) knowledge of age-related and lifetime risk; (6) knowledge of breast screening; and (7) knowledge of risk factors for breast cancer. The questionnaire was then translated to Swahili. Findings from the cognitive interview were used to revise the items for clarity. For this study report we focused on the psychometric analysis of items in exemplar domains 1 and 4 (knowledge and perceived barriers) as shown in Table 1.

**Table 1:** Domain 1 and Domain 4 of the BCAM instrument

Domain 1: Knowledge of breast cancer symptoms	Yes	No	Don’t know	Refused
Do you think a change in the position of your nipple could be a sign of breast cancer? (*Explanation: Such as pointing up or down or in a different direction to normal).* *NB: Use the labeled picture of the breast as necessary*				
Do you think pulling in of your nipple could be a sign of breast cancer? (*Explanation: Where the nipple no longer points outwards, but into the breast)*				
Do you think pain in one of your breasts could be a sign of breast cancer?				
Do you think puckering or dimpling of your breast skin could be a sign of breast cancer? (*Explanation: Like a dent or orange peel appearance.)* *NB: Use the labeled picture of the breast as necessary*				
Do you think abnormal discharge from your nipple could be a sign of breast cancer?				
Do you think bleeding from your nipple could be a sign of breast cancer?				
Do you think a lump in your breast could be a sign of breast cancer?				
Do you think a nipple rash could be a sign of breast cancer?				
Do you think if your breasts change skin color, this could be a sign of breast cancer?				
Do you think a lump under your armpit could be a sign of breast cancer?				
Do you think changes in the size of your breast could be signs of breast cancer?				
Do you think changes in the size of your nipple could be signs of breast cancer?				
Do you think changes in the shape of your breast could be signs of breast cancer? *NB: Pictures of different shapes of breasts will be provided*				
Domain 4: Barriers to screening	Yes often	Yes sometimes	No	Don’t know
Would you be too embarrassed to go and see the doctor?				
Would you be too scared to go and see the doctor?				
Would you be worried about wasting the doctor’s time?				
Would you find your doctor difficult to talk to?				
Would it be too difficult to make an appointment with the doctor?				
Would you be too busy to make time to go to the doctor?				
Would seeing the doctor be too expensive and you don’t have enough money?				
Would it be too difficult to arrange transport to the doctor’s clinic?				
Would worrying about what the doctor might find stop you from going to the doctor?				
Would not feeling confident talking about your symptom with the doctor would keep you from seeing h/m/her.				
Would significant people in your life (e.g. husband/wife, partner, sibling, relative or friend) not approve of you seeing a doctor or nurse?				
Would your doctor not understand your language?				
Would your doctor not understand your culture?				

### Cognitive interviews

These interviews took place in 6 FGDs with an average of 8 individuals per group. FGDs have been previously used as a cognitive interviewing approach to explore the understanding of items [15]. The interviews focused on comprehension of item stems and response formats for each item in the study instrument domains. This process aimed at identifying and eliminating measurement errors that might be associated with comprehension, judgment, recall, and reporting biases. FGD guide probes included components of think aloud, comprehension retrieval, judgment, and response. Cognitive interview recordings were transcribed verbatim, translated from Swahili to English, and coded for themes. Themes identified highlighted areas of concern with the scale. Revisions of the items triggered by FGD findings were made without changing the focus and meaning of the items.

### Study procedure

After changes driven by cognitive interviewing, the revised survey was administered to our target women populations in one of two languages (either English or Swahili) by trained research personnel. Written consent was obtained from all participants prior to their participation in the study. The health facility survey was administered to eligible individuals prior to screening at the respective health facilities while the household survey was administered in the household one day after the screening event. Both surveys had the same BCAM questions.

### Data analyses

For the surveys, analysis was performed using STATA version 12 special edition. Categorical variables were summarized as frequencies and the corresponding percent distributions while continuous variables, which were established to have skewed distribution, were summarized as median and corresponding inter-quartile range (IQR). Items assessing knowledge of breast cancer symptoms items were responded to with a “yes” = 1, “don’t know” = 0, “No” = −1 format. The resulting maximum score of 13 meant that participants had full knowledge of breast cancer symptoms. Scoring of barriers to breast cancer screening items was accomplished by summing item responses in a “yes often” = 2, “yes sometime” = 1, “non-response or don't know or refused” = 0 format. A maximum score of 28 was possible if the participants affirmed the highest number of perceived barriers. The test for normality was performed using the Shapiro-Wilks test. The test for associations was conducted using Pearson’s Chi Square (for categorical variables) and two-sample Wilcoxon rank sum test (for continuous and categorical variables).

Principal factoring method was performed on the items assessing knowledge of breast cancer symptoms and barriers to screening. Prior to factor analysis, Barlett’s test for sphericity as well as the Kaiser-Meyer-Olkin’s measure for sampling adequacy were done. The factors extracted were based on the Kaiser’s rule (Eigen values >1) which states that only the factors that have eigenvalues greater than one are retained for interpretation [16]. The factor loadings of the extracted factors were orthogonally rotated using the varimax method. The initial communalities were specified to be the squared multiple correlations (SMCs). After factor analysis, factor scores were computed (predicted factors) based on the factors extracted. The association between the factor scores and the categorical variables was explored using a simple linear regression model and Pearson product moment correlation for continuous variables. In addition, internal consistency of the scale was assessed using Cronbach’s alpha.

## Results

### Participant characteristics

A total of 1061 women participated in surveys, including 594 women who attended the breast cancer screening events and 467 community women who did not attend the events. Their overall median age was 34 (IQR: 26–44) years, with the majority (76%) of the women being married. Half (50%) of the women had attained more than elementary level of education, and only 62% were employed.

As shown in Table 2, women who attended the breast cancer screening events were older than the women who did not attend the events. In addition, a higher proportion of women who attended the events were married, had attained more than elementary level of education, were unemployed and had to walk longer distances to the health facilities compared to those who did not attend the events.

**Table 2:** Participants characteristics

Variables	Total (*N* = 1061)	Attended screening events N (%) or median(IQR)*or mean (SD)** (*N* = 594)	Did not attend screening events n (%) or median(IQR)* or mean (SD)** (*N* = 467)	*P* Value
Age	34(26–44)*	35(28–45)*	32(25–41)*	0.001
Marital status (Married vs. other)	809(76%)	472(79%)	337(72%)	0.006
Educational level (More than elementary vs. elementary)	527(50%)	306(52%)	221(47%)	0.175
Occupation				
Employed	201(19%)	130(22%)	71(15%)	
Self-employed	457(43%)	114(19%)	343(74%)	<0.001
Unemployed	399(38%)	347(59%)	52(11%)	
Means of transport (Walking vs. other)	259(55%)	302(51%)	259(55%)	0.142
Time required to travel to the health facility (in minutes)	30(20–45)*	30(20–60)*	30(20–30)*	0.001
Check breast for lumps (Yes vs. No)	519(49%)	332(56%)	187(40%)	<0.001
Having felt a breast lump (Yes vs. No)	125(12%)	94(16%)	31(7%)	<0.001
Having ever undergone any breast cancer screening (Yes vs. No)	109(10%)	64(11%)	45(10%)	0.573
Having been trained how to feel for a breast lump (Yes vs. No)	296(28%)	207(35%)	89(19%)	<0.001

‘-‘Analysis was not done on the respective variables

NB: Even though the median travel time to the health facility (in minutes) was similar in both groups the Wilcoxon rank-sum test assessed the dispersion between the two groups which significantly differed for the two groups

* is for median(IQR) and ** is for mean (SD)

Overall, about a half of the women had checked their breasts for lumps (Table 2). Only 12% had actually felt a lump, and 10% had previously undergone breast cancer screening. About a third of the women reported having been trained to feel their breasts for lumps. A larger percent of the women who attended the screening events had been trained to feel their breasts for lumps, had previously checked their breasts for lumps and had felt a breast lump, compared to those who did not attend the events (Table 2).

### Content validity

Cognitive testing of the original BCAM domains 1 and 4 revealed some potential biases that could have been influenced by cultural differences and the translation of items from English to Swahili. There were a number of double-barreled questions highlighted in domain 1 that explored knowledge of breast cancer symptoms. For example ‘**Do you think discharge or bleeding from your nipple could be a sign of breast cancer?’** was considered in our focus groups to be a double-barreled question because respondents were confused as to whether the interviewer was referring to any form of discharge or blood. Similarly **‘Do you think a lump or thickening in your breast could be a sign of breast cancer?’ ‘Do you think a lump or thickening under your armpit could be a sign of breast cancer?’** and **‘Do you think changes in the shape of your breast or nipple could be signs of breast cancer?’** elicited the same confusion associated with double-barreled question because of the inclusion of the conjunction ‘or’ in the statements**.** FGD participants recommended avoiding ‘or’ in such questions.

Given that it might be difficult to detect redness in dark-skinned persons, a majority of the women could not decide how to respond to the following item; **‘Do you think redness of your breast skin could be a sign of breast cancer?’** They requested clarification on whether the redness was due to peeling of the top surface of the skin, because they could not otherwise understand how dark skin could turn red. It was recommended that the statement be replaced with **‘Do you think if one of your breasts changes skin color, this could be a sign of breast cancer?’**

In addition, respondents had difficulty understanding the statement **‘Do you think changes in the shape of your breast or nipple could be signs of breast cancer?’** The concept of ‘shape’ was difficult for respondents to envision with the majority speculating that changes in breast ‘shape’ must mean that a breast enlarges or decreases in size, rather than a deformation of breast symmetry. It was recommended that a pictorial representation of changes in breast shapes be provided.

For items in domain 4 related to barriers to breast cancer screening, we were advised in FGDs to change all the statements to questions in order to avoid misunderstandings that might arise because our surveys were sometimes interviewer-administered rather than patient self-administered.

### Factor analysis

We used factor analysis as a tool to explore the patterns of item responses from our subpopulations, reasoning that groups who differed in their health-seeking behaviors (e.g. screening attendees and non-attendees) might also differ systematically in their responses to questions about breast cancer risk, presenting symptoms, and barriers to screening. The output of these analyses was used as a descriptive tool to decide whether differences in the ‘structure of knowledge and beliefs’ in our two subpopulations could be discerned.

There were, in fact, no striking differences in the factor loadings in the item responses from the groups of women who attended the screening events vs. those who did not attend the events (see Table 3 for individual item loading and overall factor Eigen values). In domain 1 (knowledge of breast cancer symptoms), one factor with eigenvalue of 1.50 emerged for the group that attended the screening events. Similarly, one factor emerged with an Eigen value of 1.76 among those who did not attend the events. These factors contained three similar items that focused on breast size and nipple changes. The factor loadings ranged from 0.65 to 0.74 among those who attended the screening events, and 0.75 to 0.80 among those who did not attend the screening events. The median factor score was 0.54 (IQR: −0.54, 0.54) with a minimum and a maximum of −2.39 and 0.66 respectively. This meant that the women had relatively high knowledge of breast cancer symptoms.

**Table 3:** Factor loadings

Item	Attended screening events		Did not attend screening events	
	Communalities	Factor loadings	Communalities	Factor loadings
Domain 1: Knowledge of breast cancer symptoms Factor 1				
Eigen value	1.50		1.76	
Do you think changes in the size of your breast could be a sign of breast cancer?	0.42	0.65	0.57	0.75
Do you think changes in the size of your nipple could be a sign of breast cancer?	0.55	0.74	0.63	0.80
Do you think changes in the shape of your breast or nipple could be signs of breast cancer?	0.53	0.73	0.56	0.75
Domain 4: Barriers to breast cancer screening				
Factor 1-Internal influences				
Eigen value	1.99		2.56	
Would you be too embarrassed to go and see the doctor?	0.60	0.77	0.66	0.81
Would you be too scared to go and see the doctor?	0.47	0.69	0.72	0.84
Would you be worried about wasting the doctor’s time?	0.71	0.84	0.59	0.76
I find the doctor difficult to talk to	0.49	0.68	0.38	0.58
Difficult to make an appointment with the doctor	0.29	0.54	0.30	0.52
Factor 2-External influences				
Eigen value	1.30		0.64	
Would your doctor not understand your language?	0.32	0.55	0.64	0.80
Would your doctor not understand your culture?	0.31	0.55	0.65	0.80

Factor analysis performed on items assessing barriers to breast cancer screening (domain 4) revealed two major factors across the study groups. Factor one included items focused on internal influences that were reported as barriers to screening with a median score of −0.162 (IQR: −0.163, −0.162) and a minimum and a maximum of −0.61 and 5.94, respectively. There were five items with factor loadings ranging from 0.54–0.84 and Eigen value of 1.99 for the group that attended the events. Similarly, among those who did not attend the events, there were five items with factor loadings ranging from 0.52–0.84 and an Eigen value of 2.56 (Table 3). On the other hand, the second factor included items focused on external influences viewed as barriers to breast cancer screening with a median score of −0.14 (IQR: −0.24, −0.14) and a minimum and a maximum of −1.03 and 5.68, respectively. Two items with loadings of 0.55 and Eigen values of above 1.30 were reported in the group that attended the events. Similarly, two items with loadings of 0.80 and an Eigen values of greater than 0.60 emerged from the group that did not attend the events.

We further explored measurement variance within the two factors (internal and external barriers) by screening uptake behaviors. Though internal knowledge influences appeared to be higher for screening attendees than non-attendees, on testing there were no statistically significant differences in perceived internal (*p* = 0.07) and external (*p* = 0.63) barriers between the two groups in factor scores.

### Reliability

A Cronbach’s alpha of 0.80 and above was reported for all the emerging factors across the two domains, except for factor two within Domain 4 that reported a score of 0.60, indicating an acceptable internal consistency. However, higher scores were reported for the group that attended the screening events in the two factors under the barriers to screening domain compared to the group that did not attend the screening events (Table 4).

**Table 4:** Internal consistency

	Among those who did not attend screening							Among those who attended screening				
Item	n	Sign	ITC	IRC	AIIC	CA	n	Sign	ITC	IRC	AIIC	CA
DOMAIN 1												
Do you think changes in the size of your breast could be a sign of breast cancer?	465	+	0.86	0.69	0.65	0.79	594	+	0.81	0.57	0.61	0.76
Do you think changes in the size of your nipple could be a sign of breast cancer?	465	+	0.88	0.73	0.59	0.74	594	+	0.85	0.65	0.51	0.67
Do you think changes in the shape of your breast or nipple could be signs of breast cancer?	465	+	0.86	0.68	0.66	0.79	594	+	0.85	0.64	0.52	0.69
Test scale					0.63	0.84					0.55	0.80
DOMAIN 4												
FACTOR 1												
Would you be too embarrassed to go and see the doctor?	467	+	0.83	0.71	0.49	0.79	594	+	0.83	0.72	0.51	0.81
Would you be too scared to go and see the doctor?	467	+	0.86	0.76	0.47	0.78	594	+	0.78	0.65	0.54	0.83
Would you be worried about wasting the doctor’s time?	467	+	0.82	0.69	0.49	0.80	594	+	0.88	0.80	0.47	0.78
I find the doctor difficult to talk to	467	+	0.73	0.57	0.55	0.83	593	+	0.78	0.63	0.56	0.83
Difficult to make an appointment with the doctor	467	+	0.69	0.51	0.58	0.85	594	+	0.71	0.55	0.59	0.85
Test scale					0.51	0.84					0.53	0.85
FACTOR 2												
Would your doctor not understand your language?	467	+	N/A	N/A	0.73	0.85	594	+	N/A	N/A	0.43	0.60
Would your doctor not understand your culture?	467	+	N/A	N/A			594	+	N/A	N/A		

*ITC* item-test correlation, *IRC*_-_ item-rest correlation, *AIIC* Average inter item correlation, *CA* Cronbach’s alpha

Item-test and item-rest correlations are only applicable when more than two variables are under assessment

### Associations between patient characteristics and knowledge of breast cancer symptoms and barriers to breast cancer screening

Following the factor analysis, Table 5 shows the association between knowledge of breast cancer symptoms and barriers to breast cancer screening constructs with patient characteristics. Women who did not attend the screening events reported higher scores on knowledge about breast cancer symptoms if they walked vs. used other means of transport. Higher scores meant that they had more knowledge about breast cancers symptoms. They were also more likely to have more knowledge about breast cancer symptoms if they had checked their breasts for lumps and had been trained. On the other hand, women who attended the screening events reported significantly lower scores for knowledge of breast cancer symptoms if they were self-employed vs. formally employed. In addition, those who walked to the health facility were more likely to report more barriers associated with external influences (Table 5).

**Table 5:** Associations between domains after factor loadings and patient characteristics

Variables	Attended screening events			Did not attend screening events		
	Knowledge of breast cancer symptoms Factor 1	Barriers to screening Factor 1-Internal influences	Barriers to screening Factor 2-External influences	Knowledge of breast cancer symptoms Factor 1	Barriers to screening Factor 1-Internal influences	Barriers to screening Factor 2-External influences
Age (per 10 years increase)	0.04(−0.01, 0.09)	0.00(−0.06, 0.05)	0.02(−0.02, 0.06)	0.04(−0.02, 0.10)	0.03(−0.03, 0.10)	0.05(−0.01, 0.11)
Married vs. others	0.14(−0.03, 0.31)	0.15(−0.04, 0.32)	0.05(−0.07,0.19)	0.09(−0.09,0.27)	−0.09(−0.28,0.10)	0.05(−0.12,0.22)
Occupation Self-employed vs. employed	**−0.23(−0.44, −0.02)**	−0.01(−0.24,0.22)	−0.13(−0.30,0.03)	−0.17(−0.42,0.08)	−0.08(−0.33,0.19)	0.08(−0.16,0.32)
Walking vs. other means of transport	−0.03(−0.17, 0.11)	0.01(−0.14,0.16)	**0.10(0.00,0.20)**	**0.28(0.12,0.44)**	0.00(−0.17,0.17)	0.03(−0.13,0.18)
Having checked breast for lumps	0.01(−0.13, 0.15)	0.03(−0.12,0.18)	−0.05(−0.15,0.06)	**0.22(0.06,0.39)**	0.02(−0.15,0.19)	−0.04(−0.20,0.11)
Having been trained how to feel for a breast lump	0.04(−0.01, 0.18)	0.04(−0.11,0.20)	−0.02(−0.13,0.09)	**0.21(0.00,0.41)**	−0.01(−0.22,0.20)	−0.17(−0.36,0.03)

The scores represent -regression coefficient (95% CI)

Statistically significant scores are in bold

## Discussion

Breast cancer is a globally important cause of morbidity and mortality not only in developed countries but also in resource-scarce sub-Saharan countries where screening and early detection programs are available [1–5, 17]. In all settings we need to have valid measures to assess knowledge of breast cancer symptoms and barriers to screening if promotion of screening and educational programming is to advance. Our findings suggest the BCAM instrument that was developed for the UK population [11] could be adapted for use in a Kenyan population. Given the socio-cultural differences between the two populations, however, a few modifications of items intended to improve the face validity and understandability of items as well as exploratory analysis to uncover internal instrument structure seemed warranted.

Cognitive testing of BCAM content highlighted a number of items that could have introduced comprehension and reporting biases. These included double-barreled questions, cultural interpretation differences and translation of items to the local language, emphasizing the importance of cross-cultural adaptation of measures. There were no significant differences in factor scores between the screening attendee and non-attendee groups suggesting that these groups may not differ in the way they perceived breast cancer symptoms and barriers to screening. We therefore believe that the BCAM scale can be used in both kinds of subpopulations.

Based on the factors that emerged in the two groups, our findings revealed that these women had a relatively high knowledge of breast cancer symptoms contrary to a similar study in the UK [11]. Unfortunately this BCAM factor in our study contained only three items focused on breast and nipple physical changes, unlike the previous study which used eleven items to characterize this domain of knowledge [11]. We also noted that items affirmed in our application of the BCAM represented symptoms of late-stage breast cancer. Previous studies have shown that women in this region present at the late-stage of the disease making it difficult to mitigate its adverse effects [4]. If we are to develop an effective tool to assess and advance breast cancer awareness in these Kenyan populations to promote screening, there is a need to incorporate additional items that comprehensively assess early-stage manifestations as well as late-stage signs of breast cancer.

Internal consistency scores on the emerging factors were found to be statistically acceptable suggesting that the scales could be consistently used to assess knowledge of breast cancer symptoms for those who did not attend the events and barriers to screening for both groups.

Overall a third of the women reported having been trained to check their breasts for lumps. It was perhaps not surprising that compared to women who attended screening, women who did not attend the screening events were more knowledgeable about symptoms of breast cancer (than those who chose to attend) and were more likely to have been previously trained on how to check their breast for lumps. It was, however, interesting that among women who did not attend the screening events, those who walked to clinic (compared to using other means of transport) were more knowledgeable about breast cancer symptoms. These observations are unexplained but could imply that those who knew a lot about the disease did not feel the need for screening, and that women who lived close to the health facility had more access to breast cancer information.

We noted that self-employed women who attended screening had significantly lower knowledge of the symptoms of breast cancer. In this region, ‘self-employment’ usually means peasant farming or vegetable vending. This group of individuals is often of a lower educational and socio-economic status, conditions that could influence their level of knowledge of breast cancer symptoms. Further studies are needed to better understand these findings.

Generally both groups of women reported minimal internal and external barriers to screening yet very few women had previously attended breast cancer screening. This suggests that uptake of breast cancer screening may be more influenced by structure or environmental factors other than personally perceived barriers.

Based on our findings we believe that providing adequate information about breast cancer symptoms, how to perform self-breast exams, and how often one should undergo breast exams by trained health providers may promote positive behavior change and enhance screening uptake across the region. It is certainly reasonable to offer re-examination services to women who have previously felt lumps in their breasts, but to maximize early detection and secondary prevention, women with no prior history of breast lumps should be motivated to attend screening events. Survey information from asymptomatic community women, such as those included in this study, may be useful in such a motivational campaign.

This study is not without limitations. The health facility-based survey targeted women who presented themselves for breast cancer screening and recruited nearly half of those in attendance to participate in the survey. Our survey personnel did not capture information on household family composition and participation rate in the community survey. In the community survey, therefore, it is not possible to measure a true population-based survey participation rate. For these reasons, biases from different participation rates may have influenced our findings. We improved the BCAM items by cognitive testing and item revision, but studies are still needed to develop appropriate measures to assess knowledge of early stages of breast cancer in this region.

## Conclusion

In conclusion, various sub-populations in western Kenya appear to differ in their propensity to participate in health-center based breast cancer screening. Educational and promotional campaigns to enhance participation in such screening will need better instrumentation for characterizing individual and population knowledge and beliefs. Our findings suggest that the BCAM instrument can be adapted for use in a Kenyan population to assess knowledge of breast cancer symptoms and barriers to screening uptake. Given the cultural differences between the UK and Kenyan populations, however, a few modifications are needed to enhance the performance of the measure. We believe that this study has taken a modest step on the journey to developing a more comprehensive, valid and reliable scale for this Kenyan population.

## Abbreviations

AMPATH: Academic model providing access to healthcare
AOI: AMPATH Oncology Institute
BCAM: Breast module of cancer awareness measure
FGD: Focus group discussions
IRB: Indiana University Institutional Review Board
IREC: Moi University Institutional Research and Ethics Committee
